# Medical Society of Kentucky

**Published:** 1853-10

**Authors:** 


### MEDICAL SOCIETY OF KENTUCKY.

The State Medical Society will meet at Lexington, on Wednesday, the 19th instant, and there is a promise of a large and spirited meeting. At the last meeting a great amount of business was provided for the year, and many reports may be expected from the Committees. The Society has already achieved a high reputation, and we trust its future transactions will add to its fame. Will not every physician of the State feel it his duty to countenance the Society, and, if possible, attend its meetings? We hope to see representatives from every quarter of the Commonwealth in Lexington, and if gentlemen will but attend once, we are sure they will find the reünions so agreeable that they will not be content to be absent from the future meetings. We promise that Louisville will be fully represented in the approaching meeting in Lexington.

---

